# Short-term environmental nitrogen dioxide exposure and neurology clinic visits for headaches, a time-series study in Wuhan, China

**DOI:** 10.1186/s12889-023-15770-0

**Published:** 2023-05-05

**Authors:** Haoyue Xu, Min Xu, Zheng JC, Fei Ye, Xiaozhou Liu, Yumin Liu, Xiaoqing Jin

**Affiliations:** 1 The Emergency Center, Zhongnan Hospital of Wuhan University, Wuhan, 430071 Hubei China; 2 The Second Clinical School of Wuhan University, Wuhan, 430071 Hubei China; 3 Department of Geriatrics, Zhongnan Hospital of Wuhan University, Wuhan, 430071 China; 4 Department of Neurology, Wuhan Central Hospital Affiliated to Tongji Medical College, Huazhong University of Science and Technology, Wuhan, 430014 Hubei China; 5 Department of Neurology, Zhongnan Hospital of Wuhan University, WuhanHubei, 430071 China

**Keywords:** Nitrogen dioxide (NO_2_), Air pollution, Headaches, Neurology clinic visits (NCVs), Time-series study

## Abstract

**Background:**

Previous studies showed the adverse impacts of air pollution on headache attacks in developed countries. However, evidence is limited to the impact of exposure to air pollutants on headache attacks. In this study, we aimed to explore the impact of nitrogen dioxide (NO_2_) exposure on neurology clinic visits (NCVs) for headache onsets.

**Methods:**

Records of NCVs for headaches, concentrations of ambient NO_2_, and meteorological variables were collected in Wuhan, China, from January 1st, 2017, to November 30th, 2019. A time-series study was conducted to investigate the short-term effects of NO_2_ exposure on daily NCVs for headaches. Stratified analyses were also computed according to season, age, and sex, and the exposure–response (E-R) curve was then plotted.

**Results:**

A total of 11,436 records of NCVs for headaches were enrolled in our study during the period. A 10-μg/m^3^ increase of ambient NO_2_ corresponded to a 3.64% elevation of daily NCVs for headaches (95%CI: 1.02%, 6.32%, *P* = 0.006). Moreover, females aged less than 50 years of age were more susceptible compared to males (4.10% vs. 2.97%, *P* = 0.007). The short-term effects of NO_2_ exposure on daily NCVs for headaches were stronger in cool seasons than in warm seasons (6.31% vs. 0.79%, *P* = 0.0009).

**Conclusion:**

Our findings highlight that short-term exposure to ambient NO_2_ positively correlated with NCVs for headaches in Wuhan, China, and the adverse effects varied by season, age, and sex.

**Supplementary Information:**

The online version contains supplementary material available at 10.1186/s12889-023-15770-0.

## Introduction

Headache is a common clinical complaint which constitutes an important cause of mortality, hospitalization, emergency room visits, and outpatient clinic service [1, 2]. Epidemiological surveys have shown that the incidence of headaches is increasing globally [3, 4]. Furthermore, according to the Global Burden of Disease study, headache is one of the most disabling symptoms worldwide [5]. Therefore, a full understanding of the risk factors for headache onsets is essential to public health. Many self-reported factors, such as weather, diet, sleep, menstruation, and stress, have been mentioned as potential triggers for headache attacks [6–9]. Recently, ambient air pollution has been proven to be positively associated with increased mortality, hospital admissions, or emergency room visits for respiratory and cardiovascular diseases [10–12]. However, the association between exposure to ambient air pollutants and headache attacks has not been well established. Several studies have demonstrated that ambient air pollution may be an important contributor to headache attacks [13, 14]. The association is biologically plausible in that air pollutants may cause oxidative stress and neurogenic inflammation, further affecting headache attacks [15]. Nevertheless, most studies have been conducted in developed countries, and these data have focused on events of hospital admissions, emergency room visits, and incidence or mortality of headaches. Data are scarce on the relationships between exposure to ambient nitrogen dioxide (NO_2_) and daily neurology clinic visits (NCVs) for headaches in developing countries.

Air pollution is a critical environmental issue, especially in developing countries. This study was conducted among residents in Wuhan, one of the twenty most polluted cities in China, with a dense population and extensive outpatient visits. This ensured adequate data on local meteorology and daily NCVs for headaches. Considering the complexity of air pollution due to different regional climates and air pollution sources, we conducted a time-series analysis on the correlation between short-term NO_2_ exposure and daily NCVs for headaches and explored the corresponding exposure–response (E-R) relationship in this study. This issue could influence decision-making in the rational allocation of medical resources and health policy-making in cities with similar emission conditions.

## Methods and materials

### Neurology clinic visits data (NCV)

Records of NCVs for headaches were extracted from the hospital information system at Zhongnan Hospital of Wuhan University between January 1st, 2017, and November 30th, 2019. Most NCVs for headaches at Zhongnan Hospital were from outpatients in the Wuchang district of Wuhan, where the hospital is located. The Wuchang district comprises 19% of the total urban population and 12% of the population of Wuhan (http://www.wuhan.gov.cn). A total of 11,436 records of NCVs with a chief complaint of headaches were enrolled as the study sample. This dataset included the dates of NCVs for headaches and related demographic information such as sex, age, and residential address of enrolled participants. To ensure the accuracy of this study, we excluded individuals who were not permanent residents of Wuhan and those who had previously visited Zhongnan Hospital for recurring headaches. Headache was diagnosed with codes G43-G44 according to the tenth version of the International Classification of Diseases (ICD-10). Due to the strict triage system in our hospital outpatient clinics, the included patients' diagnoses were almost exclusively primary headaches. The study protocol was approved by the Medical Ethics Committee of Zhongnan Hospital (IRB number: 2022142 K).

### Data of air pollution and meteorology

**Fig. 1:**
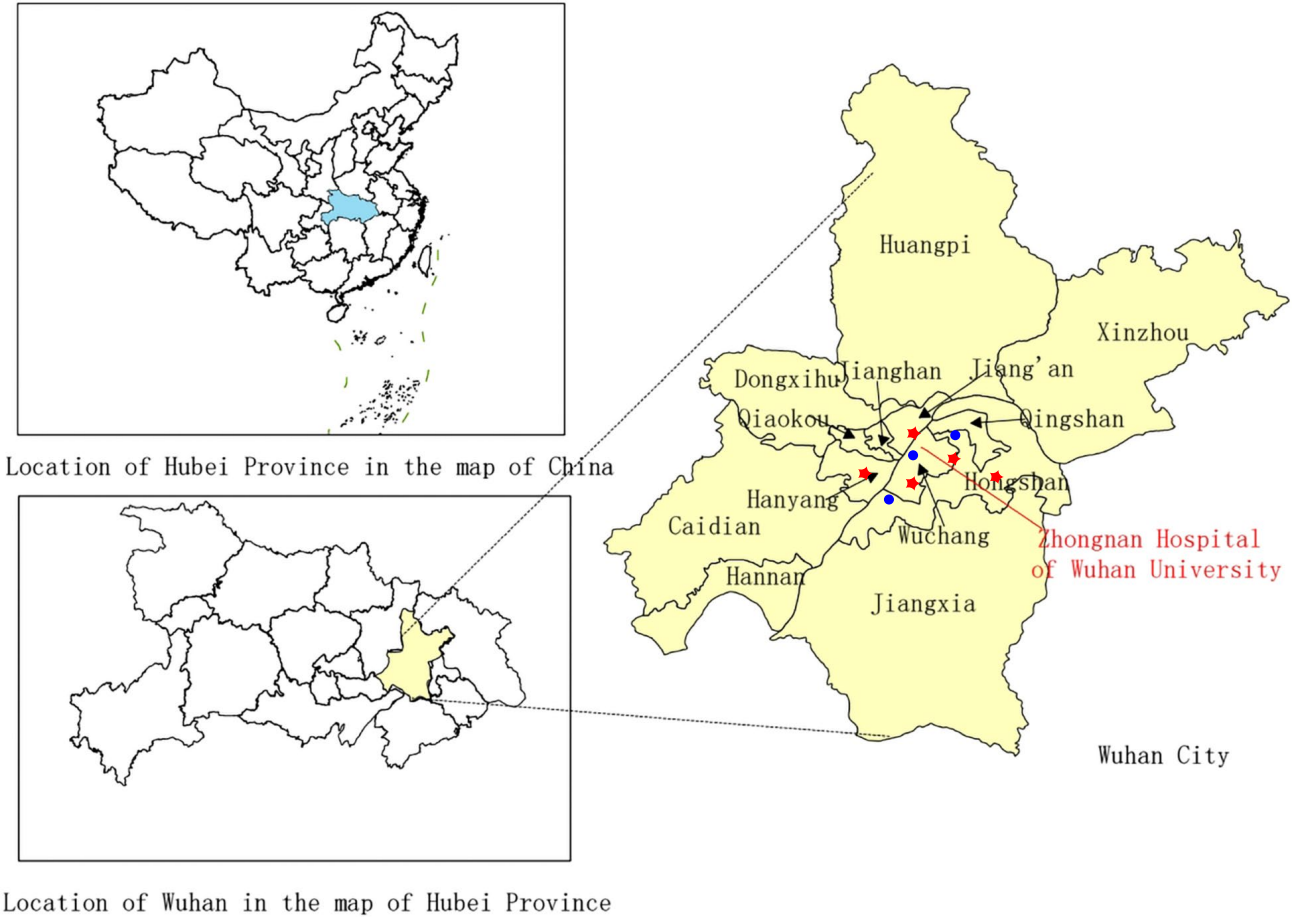
The location of Wuhan and monitoring stations. Red stars: air quality monitoring stations; Blue dots:weather stations

Daily ambient air pollution data for the study period from January 1st, 2017, to November 30th, 2019, were obtained from the Wuhan Ecological Environment Bureau website (http://hbj.wuhan.gov.cn/). The daily average concentrations of air pollutants (SO_2_, NO_2_, PM_2.5_, PM_10_, CO, and O_3_) were calculated by averaging hourly values from the fixed-site monitoring stations (Fig. 1). The daily maximum 8-h average level of O_3_ and 24-h average levels of the remaining pollutants were noted. All monitoring stations were located away from industrial, residential, and vehicular sources, ensuring the monitoring data reflects the background air condition without indeterminate interference. The daily average concentration of NO_2_ at the monitoring stations was used as a proxy for the overall NO_2_ exposure of the study population.

Meteorological data of daily average ambient temperature [°C], relative humidity [%], and barometric pressure [KPa] during the study period were acquired from the Meteorological Data Sharing Service System of the China Meteorological Administration (Beijing, China). During the study period, 0.66% of dates (7 days) were missing in the environmental and meteorological data, and we excluded these dates from our analysis.

### Statistical analyses

The data for this study were analyzed using an over-dispersed generalized additive model (GAM) to conduct a time-series analysis of the short-term effects of ambient NO_2_ on daily NCVs for headaches in Wuhan. The GAM model limits the prediction error of the dependent variable Y in various distributions, ensuring its essential role of GAM in analyzing relationships among ambient NO_2_, meteorological changes, and adverse health outcomes [16]. Data of daily NCVs for headaches followed an over-dispersed Poisson distribution, so quasi-Poisson regression was used in the GAM model. Meanwhile, several covariates were incorporated into the GAM model to control time-invariant and time-varying confounding effects. First, a natural cubic smooth function for calendar time with 7 degrees of freedom (df) per year was used to exclude long-term and seasonal trends [17, 18]. Second, natural smoothing functions of daily average ambient temperature (6 df), relative humidity (3 df), and barometric pressure (3 df) were included to control the non-linear confounding effect of meteorological factors [19]. Third, other covariates, such as public holidays (Holidays) and days of the week (DOW), were adjusted as dummy variables in the GAM model [20].

The GAM model was described as follows:

$$logE\left(Yt\right)={\beta Z}_t+DOW+ns\left(time,\;df\right)+ns\left(temperature,\;\mathit6\right)+ns\left(humidity,\;\mathit3\right)+ns\left(pressure,\;\mathit3\right)+intercept$$

In the above model, *E(Yt)* represents the estimated daily NCVs for headaches at day t; *β* represents the log-relative rate of NCVs for headaches correlated with per increased air pollutant unit; *Z*_*t*_ represents the average concentration of NO_2_ at day* t*; *DOW* is a dummy variable for days of the week; and *ns* refers to the natural cubic regression smooth function. Two different lag time constructions were conducted for short-term NO_2_ exposure: the single-day lags algorithm (lag0 to lag7) and the multi-day algorithm of moving average lags (lag0-1 to lag0-7). Then three statistics methods of Akaike Information Criterion (AIC), Generalized Cross Validation (GCV), and Partial Autocorrelation Function (PACF) were applied to choose the optimal lag structure. To examine the non-linear correlation and the presence of a threshold concentration of NO_2_, we plotted the exposure–response (E-R) relationship curve between short-term exposure to NO_2_ and daily NCVs for headaches by replacing the linear term of NO_2_ with a natural spline function of 3df to the above model.

Three sensitivity analyses were then performed to check the stability of this model. First, an alternative 4–10 df per year was selected for the smoothness of the temporal trend. Second, sensitivity analyses were conducted with different numbers of df in the natural cubic splines of temperature, relative humidity, and pressure. Third, a two-pollutant model was used to assess the robustness of effect estimates after adjusting for co-pollutants with a correlation coefficient inferior to 0.7.

Three stratification analyses according to season (warm: April to September; cool: October to March), age (< 50 years; ≥ 50 years), and sex (females; males) were then conducted to explore the discrepant associations in different subgroups of the study population and different seasons. The age stratification was based on an epidemiological survey conducted throughout China, which showed that the incidence of primary headaches increased with age, peaking at age 50; after that, the incidence declined [21].

All statistical analyses were performed in R software (version 4.1.0) using the MGCV package. Effects were described as percent change and the corresponding 95% CI in daily NCVs for headaches per 10 μg/m^3^ increase of ambient NO_2_ exposure. The statistical significance of differences between the strata effect estimates was computed by calculating 95% confidence intervals as $\left({\widehat{Q}}_{1}-{\widehat{Q}}_{2}\right)\pm 1.96\sqrt{{\left(S{\widehat{E}}_{1}\right)}^{2} + {\left(S{\widehat{E}}_{2}\right)}^{2}}$, where ${\widehat{Q}}_{1}$ and ${\widehat{Q}}_{2}$ are the estimates for two categories, and SÊ_1_ and SÊ_2_ represent the corresponding standard errors. A two-tailed *p* < 0.05 was used to determine the statistical significance.

## Result

A total of 11,436 records of NCVs for headaches were enrolled in the present study from the hospital information system of Zhongnan Hospital, Wuhan University, for the study period between January 1st, 2017, and November 30th, 2019. According to statistics, the included patients were diagnosed with primary headaches. As shown in Table 1, females accounted for 59.6% of NCVs for headaches, while individuals over the age of 50 represented 51.0% of the total NCVs for headaches. The incidence of daily NCVs for headaches was slightly higher in warm seasons than in cool seasons (52.9% vs. 47.1%). Additionally, the daily average concentration of ambient NO_2_ during the study period in Wuhan was 46.22 μg/m^3^, which exceeded Chinese secondary ambient air quality standards. The daily average ambient temperature, relative humidity, and barometric pressure were 17.70 °C, 78.76%, and 101.51 kPa, respectively.

**Table 1:** The summary of daily NO_2_, weather conditions, and daily neurology clinic visit for headaches (*N* = 11,436) during our study period (January 1st, 2017 to November 30th, 2019)

	Mean	SD	Min	P25	Median	P75	Max
**Air pollutant concentration (μg/m3)**^a^							
NO_2_	46.22	19.53	13	31	42.72	59	119
**Meteorological measures**							
Temperature (°C)	17.70	9.38	-3.8	9.58	18.5	25.8	33.9
Humidity (%)	78.76	10.38	41	72	79	87	100
Pressure (KPa)	101.51	0.99	99.54	100.6	101.55	102.26	104.08
**No. Of neurology clinic visit for headache**	11	6	0	7	11	14	38
**Season(N)**							
Warm^b^	11	6	0	7	11	15	38
Cool^c^	10	5	0	7	10	14	29
**Gender(N)**							
Male	4	3	0	2	4	6	22
Female	6	4	0	4	6	9	23
**Age(N)**							
< 50	5	3	0	3	5	7	19
≥ 50	5	3	0	3	5	8	19

*Abbreviations*: *SD* Standard deviation, *P25* 25th percentile, *P75* 75th percentile, *NO*_*2*_ Nitrogen dioxide

^a^24-hour average for NO_2_

^b^Warm season: from April to September

^c^Cool season: from October to March

**Fig. 2:**
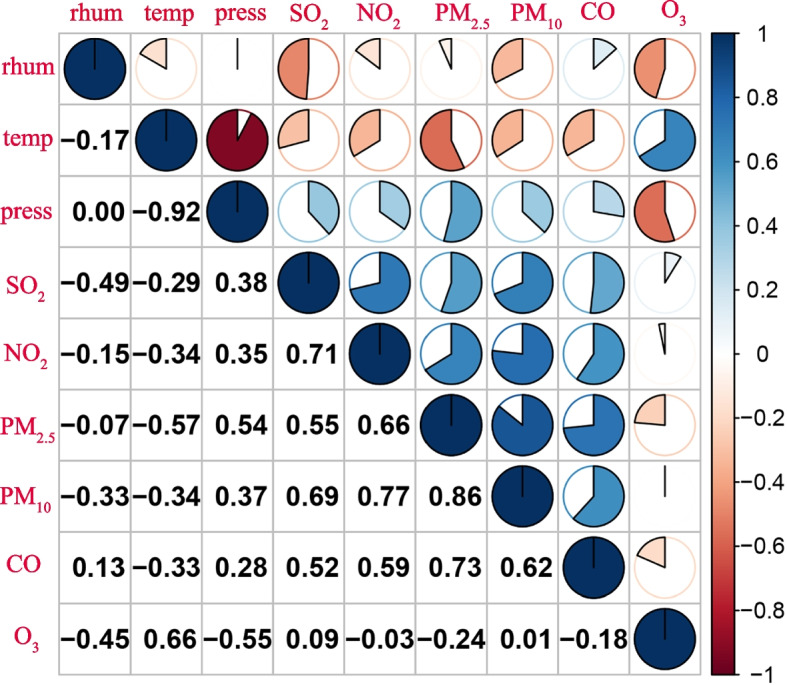
Spearman correlations among exposure variables in Wuhan, China (2017–2019). temp, temperature; rhum, relative humidity

Figure 2 showed that the daily average concentration of ambient NO_2_ during the study period was positively correlated with that of ambient SO_2_, PM_2.5_, PM_10_, and CO, with Spearman's coefficients ranging from 0.59 to 0.77. Conversely, the daily average level of ambient NO_2_ showed a negative correlation with the daily average levels of O_3_ ( Spearman's coefficient:-0.03). Additionally, ambient temperature (Spearman's coefficients: -0.34) and relative humidity (Spearman's coefficients: -0.15) were negatively associated with the daily average concentration of ambient NO_2_. On the other hand, barometric pressure was positively correlated with the daily average concentration of ambient NO_2_, with a Spearman's coefficient of 0.35.

**Fig. 3:**
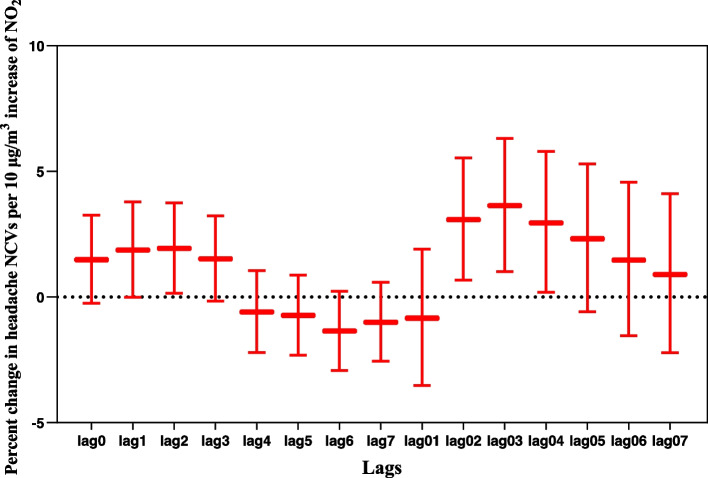
Percentage change in NCVs for headaches when NO_2_ concentrations increased 10 μg/m^3^ using different lag structures. Horizontal coordinate: lag structures (lag0 to lag7, and lag01 to lag07). Vertical coordinate: percentage (%) change in NCVs for headaches (mean and 95%CI) associated with a 10 μg/m^3^ increase of NO_2_ concentrations

Figure 3 demonstrated the percentage changes of daily NCVs for headaches (mean and 95% CI) per 10 μg/m^3^ increase of ambient NO_2_, using algorithms of the single-day lags (lag1-lag7) and multi-day moving average lags (lag01-lag07). Based on the model fitting statistics, lag03 of NO_2_ exposure was chosen as the optimal lag structure with the smallest AIC/GCV/PACF values. Our data showed that the daily average level of ambient NO_2_ was positively associated with daily NCVs for headaches. Specifically, a 10 μg/m^3^ increase of ambient NO_2_ corresponded to a 3.64% elevation of daily NCVs for headaches (95%CI: 1.02%, 6.32%) (Additional file 1).

Sensitivity analyses conducted using an alternative 4–10 df per year demonstrated that short-term effects of NO_2_ exposure remained significant after adjusting for temporal smoothness (Additional file 2). And the results remained robust when using different numbers of df in the natural cubic splines of temperature, relative humidity, and pressure (Additional file 3). Furthermore, after incorporating co-pollutants with Spearman's correlation coefficients lower than 0.7 into the two-pollutants model, there was still a significant association between short-term NO_2_ exposure and daily NCVs for headaches (Table 2).

**Table 2:** Percent change (%, mean and 95% CI) in NCVs for headaches in two-pollutant models

Two-pollutants		Percent change
NO_2_^a^	-	3.64 (1.02, 6.32)*
	+ PM_2.5_	3.48 (0.86, 6.18)*
	+ CO	3.42 (0.76,6.14)*
	+ O_3_	3.56 (0.95, 6.24)*

^*^ *p* < 0.05

^a^Moving average of lag 03 was used for NO_2_

Table 3 illustrates the percentage changes of daily NCVs for headaches (mean and 95% CI) per 10 μg/m^3^ increase of ambient NO_2_, stratified by season, age, and sex. Short-term exposure to NO_2_ showed a positive correlation with daily NCVs for headaches throughout the year, and this correlation was statistically significant in cool seasons (6.31%, 95% CI: 2.57%, 10.19%) but insignificant in warm seasons (0.79%, 95% CI: -3.47%-5.24%). Younger individuals under 50 years of age were slightly more susceptible than those aged more than 50 years old (3.73%, 95% CI: 0.39–7.18% vs. 3.60%, 95% CI: 0.36%-6.94%). Additionally, the correlation between ambient NO_2_ exposure and daily NCVs for headaches was significantly stronger among females than males (4.10% vs. 2.97%).

**Table 3:** Percent change (95% CI) in headache NCVs with a 10 μg/m^3^ increase in air pollutant concentrations by season, gender and age in Wuhan, China

Pollutants	Season		Gender		Age	
	Cool	Warm	Female	Male	Younger	Elder
NO_2_^a^	6.31 (2.57,10.19) *	0.79 (-3.47, 5.24)	4.10 (1.10,7.20) *	2.97 (-0.60,6.67)	3.73 (0.39,7.18) *	3.60 (0.36,6.94) *

^*^p < 0.05

^a^Moving average of lag 03 was used for NO_2_

**Fig. 4:**
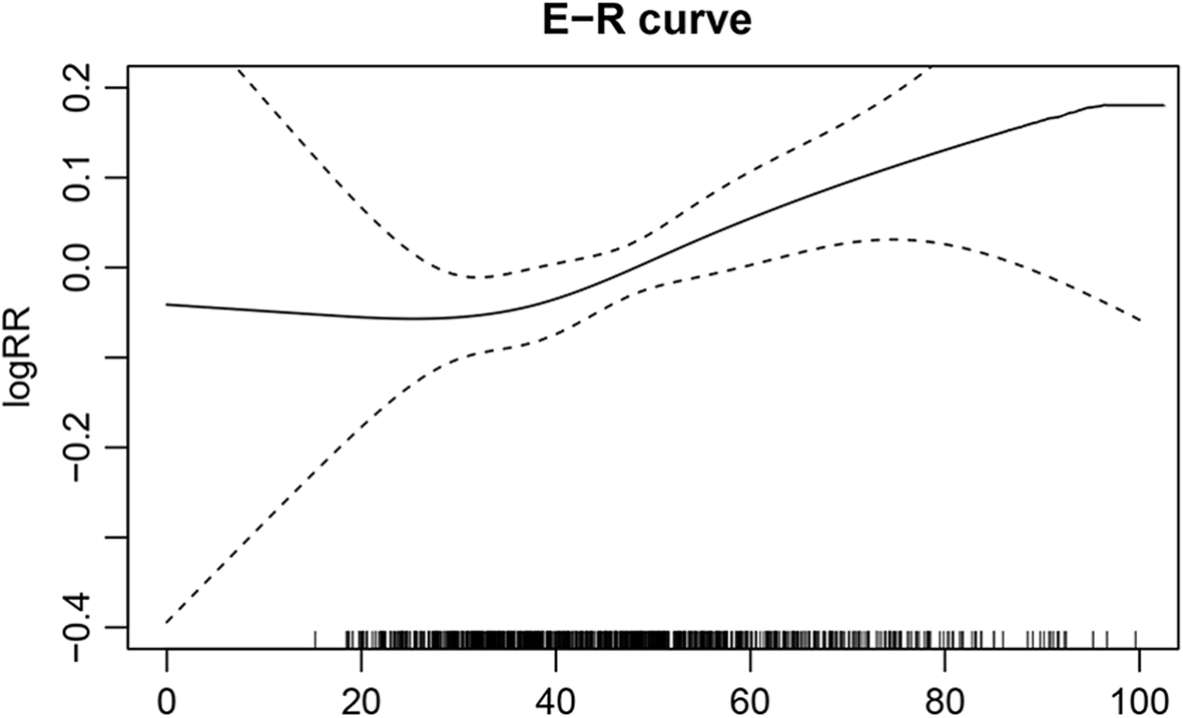
The exposure–response relationship curve of NO_2_

Figure 4 graphically illustrates the exposure–response (E-R) curve of the relationship between short-term exposure to ambient NO_2_ and daily NCVs for headaches. The E-R graph showed an S-shaped curve with a sharp rise in the interval of 40-80 μg/m^3^, and it levels off at more than 80 μg/m^3^.

## Discussion

Our study demonstrated a significant correlation between short-term exposure to NO_2_ and increased daily NCVs for headaches (primary headaches), which remained robust after adjusting for co-pollutants. Furthermore, the correlation was stronger in patients under 50 years of age and females. The acute effect of NO_2_ on headache patients was more substantial in cool seasons than in warm seasons. This analysis added to the limited evidence on the effect of ambient NO_2_ exposure on the incidence of primary headaches in developing countries.

In this study, short-term exposure to NO_2_ was positively correlated with increased daily NCVs for headaches (primary headaches), which is generally consistent with previous studies [22–25]. NO_2_ is asphyxiating odorous gas characterized as one of the environmental irritants. Inhaled NO_2_ acts directly on the olfactory nerve via the olfactory epithelium, causing damage and dysfunction of the olfactory system (e.g. olfactory loss). Simultaneously, transient receptor subfamily vanilloid 1 (TRPV1) and subfamily ankyrin 1 (TRAP1), which are highly expressed in the olfactory and trigeminal nerve endings, are activated provoking neurological susceptibilities through oxidative stress and systemic inflammation [26, 27]. TRPV1 and TRAP1 are crucial in the pathophysiological process of pain sensation and inflammation [28, 29]. The stimulated TRPV1 and TRAP1 receptors contribute to central sensitization, facilitating the transmembrane entry of calcium ions into neurons [27, 30]. Over-activated TRPV1 and TRAP1 receptors lead to calcium overload in neurons, resulting in neuron apoptosis [27]. Moreover, inhaled NO_2_ can damage the blood–brain barrier and promote distal neurogenic inflammation by releasing Substance P or other neuropeptides [31–33]. Kunkler reported that exposure to environmental irritants (e.g. air pollutants) induced rats' migraines by stimulating TRPA1 receptors in nasal epithelial cells [34, 35]. The above evidence provides some explanations for the increased daily NCVs for primary headaches related to short-term exposure to ambient NO_2_ from a cellular level.

Epidemiologically, two studies conducted in Italy and Chile revealed that high concentrations of NO_2_ exposure were associated with elevated frequency, severity, and relative risks of headache attacks [23, 36]. Moreover, a time-stratified case-crossover study in Korea demonstrated that the association between ambient NO_2_ exposure and emergency room visits for migraines was significantly stronger than other air pollutants [37]. These findings provide insights into the adverse effects of ambient NO_2_ exposure on headache attacks.

It is essential for a public health assessment to plot the E-R curve and figure out the threshold concentrations of each air pollutant. In our study, the E-R relation graph presented an S-shaped curve, and the threshold level of NO_2_ triggering the increase of daily NCVs for primary headaches was 40 μg/m^3^, which was lower than the standard set by the Chinese ambient air quality standards-class I (GB3095-2012). However, the S-shaped E-R curve gradually flattened out at higher concentrations, which might be related to the “harvest effect” in the susceptible populations. People who are vulnerable to NO_2_ might have already been affected and hospitalized before concentrations of NO_2_ reach high levels [38]. Although the E-R relationship can be confounded by different factors such as air pollution mixtures, meteorological conditions, and the health status of the study population, our findings remain useful in health policy-making and determining the cutoff value of contamination substances. It is essential to take potentially effective measures to control ambient NO_2_ concentrations to reduce NO_2_-associated primary headaches.

Our study further demonstrated that the association between short-term exposure to NO_2_ and daily NCVs for headaches varied according to season, age, and sex. First, our data showed that acute adverse effects of NO_2_ exposure on daily NCVs for headaches in cool seasons were about eight times as high as in warm seasons. Conversely, previous studies reported that headache attacks were influenced by temperature with an occurrence tendency mainly in warmer seasons [13, 37, 39]. This inconsistency might be associated with unfavorable winter meteorological conditions of Wuhan, where it is difficult for the dispersion of NO_2_ in weak winter monsoons [40, 41]. Furthermore, burning crop residues and the oxidation of atmospheric ammonia from agricultural production also increase NO_2_ concentrations in Wuhan between October and March [40, 42]. And excessive NO_2_ might weaken the effect of temperature on headache attacks. Second, our data also showed that females were slightly more vulnerable to NO_2_ exposure regarding daily NCVs for headaches. This susceptibility might be related to fluctuating levels of estrogen in females. Studies showed that estrogen could influence the physiological function of cerebrovascular endothelium and neuronal excitability by interaction with 5-hydroxytryptamine and norepinephrine, which might play an important role in headache attacks [43–46]. Third, our data further demonstrated individuals under 50 years old presented more frequently in NCVs for primary headaches after short-term exposure to NO_2_. However, one study conducted in Canada showed reverse age distribution concerning the influence of NO_2_ on emergency department visits for migraines [39]. There were several possible explanations speculated for this argument. First, young people have more opportunities for outdoor activities with more exposure to high levels of NO_2_, especially during peak traffic hours. Also, primary headaches presented more frequently in neurology clinic visits instead of emergency department visits. Emergency department visits are responsible for severe headaches secondary to intracranial diseases, such as cerebrovascular strokes, which happen more frequently in elders [47]. These findings are important for health-policy making to get real-world evidence of risk stratification in extremely pollutant environments.

Our study has several limitations. First, we used average concentrations of NO_2_ measured by stationary site monitoring to represent individual exposures, which may lead to inevitable exposure misclassification. Second, although we have considered some possible confounding effects of air pollutants (PM_2.5_, CO, and O_3_) and meteorological factors (temperature, relative humidity, and pressure), there may be other factors that affect the formation of headaches and impair a person's tolerance to NO_2_, such as pre-existing diseases and unhealthy factors. Third, we had highly accurate information on the date of outpatient visits but not the onset date of headache symptoms. Hence, we could not control the interference of other factors before evaluation. Fourth, our study was conducted in a highly polluted city, and the generalizability of our findings to other cities or countries with different levels and sources of air pollution may be limited. Therefore, further studies are needed to confirm our results, and molecular biology or animal experiments are necessary to explore the exact mechanisms between NO_2_ and headache attacks.

## Conclusions

Our study demonstrated that short-term exposure to ambient NO_2_ significantly correlated with increased daily NCVs for headaches in Wuhan, China. This correlation was significantly stronger in cool seasons, females, and individuals under 50. Our findings have important implications for local public and environmental strategy and policy in cities with similar emission conditions.

## Supplementary Information

**Additional file 1.** Percent change (mean and 95% CI) in NCVs for headaches associated with a 10-μg/m^3^ increase in concentrations of NO_2_ using different lag structures.

**Additional file 2.** Percentage change (mean and 95%CI) in NCVs for headache associated with a 10 μg/m3 increase in concentrations of NO2 at lag03 using different degrees of freedom per day.

**Additional file 3.** Percentage changed (mean and 95% CI) in NCVs fir headaches associated with a 10 µg/m^3^ increase in consentrations of NO2 at lag03 using different df in the natural cubic splines of temperature, humidity, and pressure.

**Additional file 4.** The meteorological introduction and geographical description of Wuhan.

**Additional file 5. **

## Data Availability

The datasets used and/or analysed during the current study are available from the corresponding author upon reasonable request.
