# Supplementary material for: Short-term environmental nitrogen dioxide exposure and neurology clinic visits for headaches, a time-series study in Wuhan, China
Source: BMC Public Health. 2023 May 5;23:828. doi: 10.1186/s12889-023-15770-0 (PMC10161479; doi:10.1186/s12889-023-15770-0)
Supplement: Supplementary file 1 — Additional file 1. Percent change (mean and 95% CI) in NCVs for headaches associated with a 10-μg/m3 increase in concentrations of NO2 using different lag structures. [file 12889_2023_15770_MOESM1_ESM.docx]

**Additional file 1** Percent change (mean and 95% CI) in NCVs for headaches associated with a 10-μg/m^3^ increase in concentrations of NO_2_ using different lag structures.

| Lag | NO_2_ |
| --- | --- |
| 0 | 1.49 (-0.25,3.26) |
| 1 | 1.87 (-0.01, 3.79) |
| 2 | **1.94 (0.15, 3.75)*** |
| 3 | 1.52 (-0.16,3.23) |
| 4 | -0.59 (-2.20,1.05) |
| 5 | -0.73 (-2.31,0.87) |
| 6 | -1.35 (-2.92,0.23) |
| 7 | -1.00 (-2.55,0.58) |
| 01^a^ | -0.84 (-3.51, 1.91) |
| 02 ^a^ | **3.08 (0.68,5.53)*** |
| 03 ^a^ | **3.64 (1.02,6.32)*** |
| 04 ^a^ | **2.95 (0.19,5.80)*** |
| 05 ^a^ | 2.32 (-0.58,5.30) |
| 06 ^a^ | 1.47(-1.54,4.57) |
| 07 ^a^ | 0.90 (-2.22,4.12) |

* p<0.05.

^a^ Lag 01(02,03,04,05,06,07) the moving average concentrations on the present day and previous 1(2,3,4,5,6,7) day.
