# Supplementary material for: Short-term environmental nitrogen dioxide exposure and neurology clinic visits for headaches, a time-series study in Wuhan, China
Source: BMC Public Health. 2023 May 5;23:828. doi: 10.1186/s12889-023-15770-0 (PMC10161479; doi:10.1186/s12889-023-15770-0)
Supplement: Supplementary file 3 — Additional file 3. Percentage changed (mean and 95% CI) in NCVs fir headaches associated with a 10 µg/m3 increase in consentrations of NO2 at lag03 using different df in the natural cubic splines of temperature, humidity, and pressure. [file 12889_2023_15770_MOESM3_ESM.pdf]

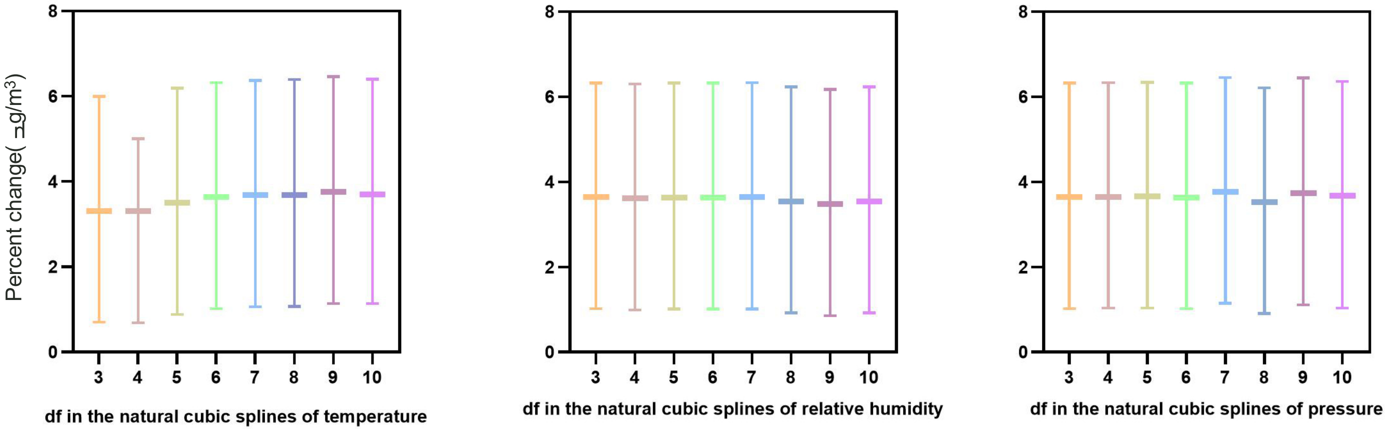

Additional file 3. Percentage change (mean and 95%CI) in NCVs for headaches associated with a 10 μg/m3 increase in concentrations of NO2 at lag03 using different df in the natural cubic splines of temperature, humidity, and pressure
