# Supplementary material for: Short-term environmental nitrogen dioxide exposure and neurology clinic visits for headaches, a time-series study in Wuhan, China
Source: BMC Public Health. 2023 May 5;23:828. doi: 10.1186/s12889-023-15770-0 (PMC10161479; doi:10.1186/s12889-023-15770-0)
Supplement: Supplementary file 4 — Additional file 4. The meteorological introduction and geographical description of Wuhan. [file 12889_2023_15770_MOESM4_ESM.docx]

**Additional file 4**: The meteorological introduction and geographical description of Wuhan

Wuhan is a large metropolitan city in central China, located at latitude 30°35'N and longitude 114°17'E, with a population over 10 million. It comprises seven central districts and six suburban/rural districts (Fig.1), covering an area of approximately 8569.15 km^2^ (http://www.wuhan.gov.cn). Wuhan has a humid monsoon subtropical climate, characterized by hot and wet summers, and cold and dry winters. The average ambient temperature in Wuhan is 30.3 °C in July and 4.4 °C in January. As an essential industrial and transport hub of China, the main sources of air pollution in Wuhan are vehicle exhaust and industrial emissions.
